# Novel dummy molecularly imprinted polymer for simultaneous solid-phase extraction of stanozolol metabolites from urine

**DOI:** 10.1007/s00216-024-05285-x

**Published:** 2024-04-25

**Authors:** Yomna G. Farag, Rasha S. Hanafi, Mennatallah A. Hammam

**Affiliations:** 1 Pharmaceutical Chemistry Department, Faculty of Pharmacy and Biotechnology, German University in Cairo, Cairo, 11835 Egypt; 2 Department of Chemistry, School of Life and Medical Sciences, University of Hertfordshire Hosted By Global Academic Foundation, New Administrative Capital, Cairo, 4813001 Egypt

**Keywords:** Bulk polymerization, Molecularly imprinted polymer, Solid-phase extraction, Stanozolol metabolites, UHPLC-MS/MS

## Abstract

Stanozolol, a synthetic derivative of testosterone, is one of the common doping drugs among athletes and bodybuilders. It is metabolized to a large extent and metabolites are detected in urine for a longer duration than the parent compound. In this study, a novel dummy molecularly imprinted polymer (DMIP) is developed as a sorbent for solid-phase extraction of stanozolol metabolites from spiked human urine samples. The optimized DMIP is composed of stanozolol as the dummy template, methacrylic acid as the functional monomer, and ethylene glycol dimethacrylate as the cross-linker in a ratio of 1:10:80. The extracted analytes were quantitively determined using a newly developed and validated ultrahigh-performance liquid chromatography tandem mass spectrometry method, where the limits of detection and quantitation were 0.91 and 1.81 ng mL^−1^, respectively, fulfilling the minimum required performance limit decided on by the World Anti-Doping Agency. The mean percentage extraction recoveries for 3’-hydroxystanozolol, 4β-hydroxystanozolol, and 16β-hydroxystanozolol are 97.80% ± 13.80, 83.16% ± 7.50, and 69.98% ± 2.02, respectively. As such, the developed DMISPE can serve as an efficient cost-effective tool for doping and regulatory agencies for simultaneous clean-up of the stanozolol metabolites prior to their quantification.

**Graphical abstract:**

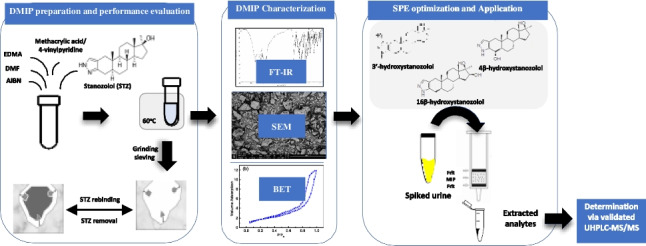

**Supplementary information:**

The online version contains supplementary material available at 10.1007/s00216-024-05285-x.

## Introduction

**Fig. 1:**
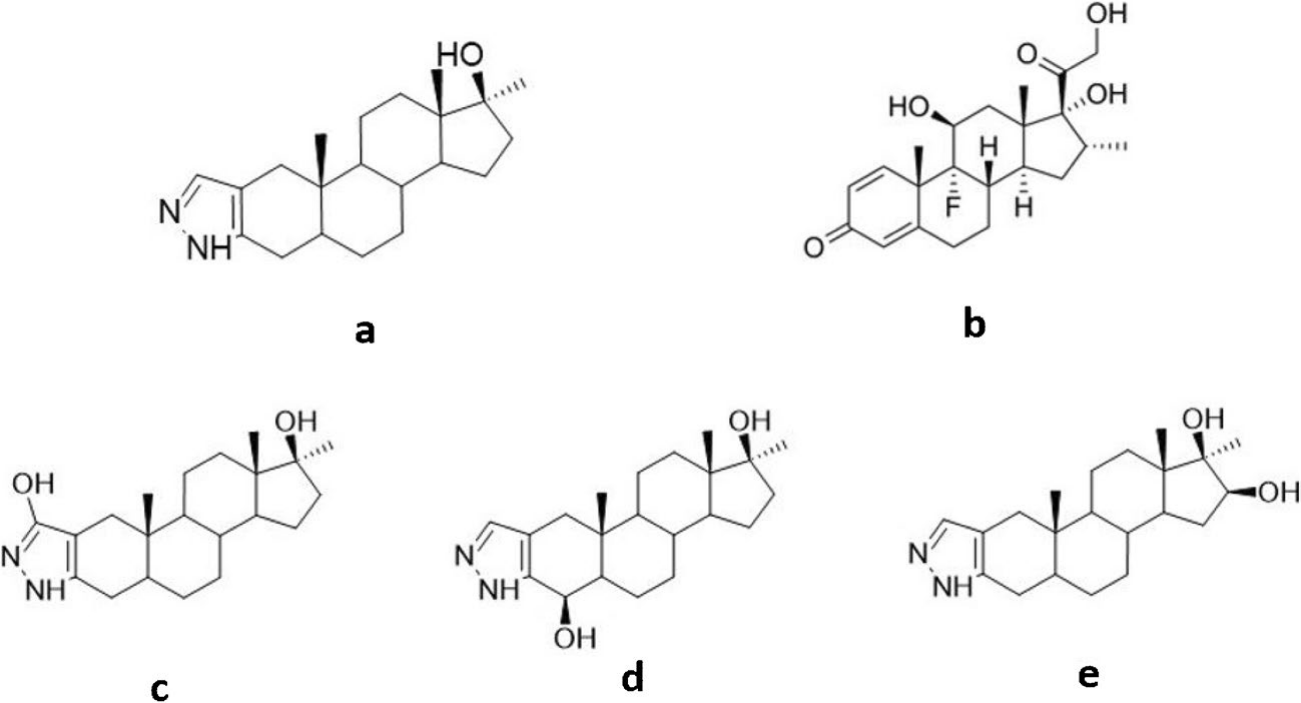
Structures of **a** STZ, **b** dexamethasone (internal standard), **c** 3’-OHSTZ, **d** 4β-OHSTZ, and **e** 16β-OHSTZ

According to the statistical report published by the World Anti-Doping Agency (WADA) in 2021, anabolic androgenic steroids (AASs) are the most abused class of doping drugs in sports [1]. Stanozolol (STZ), known in the market as *Winstrol*, is one of the most frequently used synthetic AASs among athletes and bodybuilders. It has been developed in an attempt to separate the anabolic and androgenic actions of testosterone. Unlike the common structure of all AASs, STZ and its metabolites contain a pyrazole ring attached to the steroidal A-ring. This structural modification made the extraction and isolation of STZ from bio-matrices more challenging. In addition, it resulted in a more extensive hepatic metabolism leading to minimal excretion of the parent drug in the urine [2]. As a result, the focus became the development of analytical methods for the detection of its urinary metabolites. The main metabolites of STZ are the glucuronides of 3’-hydroxystanozolol (3’-OHSTZ), 4β-hydroxystanozolol (4β-OHSTZ), and 16β-hydroxystanozolol (16β-OHSTZ) (Fig. 1) [3].

Like most anabolic steroids, STZ and its metabolites have poor gas chromatographic behavior and are difficult to derivatize [4]. Although gas chromatography tandem mass spectrometry (GC–MS/MS) [3, 5] and isotope dilution-mass fragmentography [6] were reported for the determination of STZ in urine after derivatization, liquid chromatography tandem mass spectrometry (LC–MS/MS) remains the technique of choice for determination of steroidal metabolites, attributed to its wide applicability for detection and trace analysis of the hydroxylated metabolites.

To ensure harmonized implementation of the detection protocol for the presence of prohibited substances in all doping control laboratories, WADA established a minimum detection capability for testing methods “*minimum required performance limits (MRPL).*” The MRPL for STZ is 2 ng mL^−1^ [7] and 1 ng mL^−1^ for its metabolites [8]. Hence, it became crucial to strengthen the efficiency of the sample pre-treatment methods to improve sensitivity for these doping drugs. Different sample pre-treatment techniques for STZ were reported in the literature, varying from solid-phase extraction (SPE), and liquid–liquid extraction (LLE) [9] to immunoaffinity chromatography [5]. However, the identification of STZ and its main metabolites using any of these pre-treatment methods proved to be problematic due to the interfering effect of the matrices [4].

Molecular imprinting is a process of designing synthetic polymers with predetermined specificity and selectivity for the target analyte. These molecularly imprinted polymers (MIPs) are synthesized by assembling the functional monomers (FM) around the template (T), followed by polymerization in the presence of a suitable cross-linker (CL), initiator, and porogen. The imprinted molecule is then washed out of the highly cross-linked polymer resulting in specific binding sites complementary in shape, size, and functionality to that of the template and capable of rebinding it. Using MIP as a sorbent in SPE allows for selective preconcentration of the analyte and removal of the interferents. On top of that, its reusability, low cost of preparation, and stability to harsh conditions make it advantageous over other sample pre-treatment methods such as solvent extraction and immunoaffinity-based methods [10]. The use of dummy MIPs (DMIPs) has become a choice of interest to avoid inaccurate quantitation of the analyte in real samples due to bleeding or leaching of the unwashed template remnants into the sample matrix. DMIPs are prepared using an analog molecule of the target analyte as a dummy template (DT) [11]. To the best of our knowledge, no MISPE method has been reported for the isolation of STZ metabolites from human urine using STZ as a DT. The presented study describes the development of a novel MIP using STZ as DT. The developed DMIP is used as a sorbent in the SPE cartridge for the simultaneous extraction of the STZ metabolites from spiked human urine samples prior to their quantitation using the newly developed UHPLC-MS/MS method.

## Materials and methods

### Chemicals and reagents

STZ, 3’-OHSTZ, 4β-OHSTZ, 16β-OHSTZ, acetonitrile (ACN), methanol (MeOH), 4-vinylpyridine (4-VP), methacrylic acid (MAA), ethylene glycol dimethacrylate (EGDMA), 2–2′-azoisobutyronitrile (AIBN), and formic acid (FA) were purchased from Sigma-Aldrich (Germany). Dexamethasone (DEXA), the internal standard (IS), was purchased from the Egyptian International Pharmaceutical Industries Company–EIPICO (Egypt). Anhydrous sodium acetate was purchased from El-Nasr Pharmaceutical Chemicals CO. (Egypt). Glacial acetic acid (AcOH) and dimethylformamide (DMF) were obtained from Pioneers for Chemicals (Egypt). Free fresh urine samples were obtained from one Egyptian athletic volunteer from the laboratory staff.

### Equipment and instrumentation

SPE procedures were carried out using a Visi-1 Single SPE Tube Processor purchased from Sigma-Aldrich (Germany). pH measurements were done using Jenway pH meter 3310 (UK). Ultrapure water was prepared using the Stakpure Pure water system OMNIATYPE1, Stakpure GmbH (Germany). Fourier transform infrared (FTIR) spectra were recorded using Thermo Scientific*™* Nicolet*™* iS50 FTIR spectrometer (Germany)*.* The surface area, pore volume, and pore size of the polymers were calculated using Brunauer–Emmett–Teller (BET) and Barret-Joyner-Halenda (BJH) methods via NOVAtouch® LX4 Quantachrome (USA). Surface morphology was examined using the JEOL JSM*-*6360LA microscope for field-emission scanning electron microscopy (SEM) (Japan). All the analytical measurements were carried out on ACQUITY UPLC H-Class system that is composed of Xevo™ TQD triple-quadrupole tandem mass analyzer with an electrospray ionization (ESI) interface, Mass Lynx 4.1 software, and target Lynx quantification program, all provided by Waters Corp. (USA). Acquity UPLC BEH C18 column (1.7 µm, 100 mm × 2.1 mm) was used to separate the target analytes (Waters, Wexford, Ireland).

### Standard stock solutions and buffer preparation

Acetate buffer (25 mM) was prepared by dissolving 100.0 mg of anhydrous sodium acetate (C_2_H_3_NaO_2_) in 50 mL of ultrapure water. The buffer pH was adjusted using AcOH at 1.0 and 4.0. Standard stock solution of STZ (5 µg mL^−1^) was prepared in DMF/MeOH/H_2_O (0.5/0.5/99, v/v/v), while stock solutions of 3’-OHSTZ, 4β-OHSTZ, 16β-OHSTZ (1 µg mL^−1^), and DEXA (5 µg mL^−1^) were prepared in MeOH. Standard working solutions were prepared by proper dilution of the standard stock solutions in MeOH.

### DMIP preparation

**Fig. 2:**
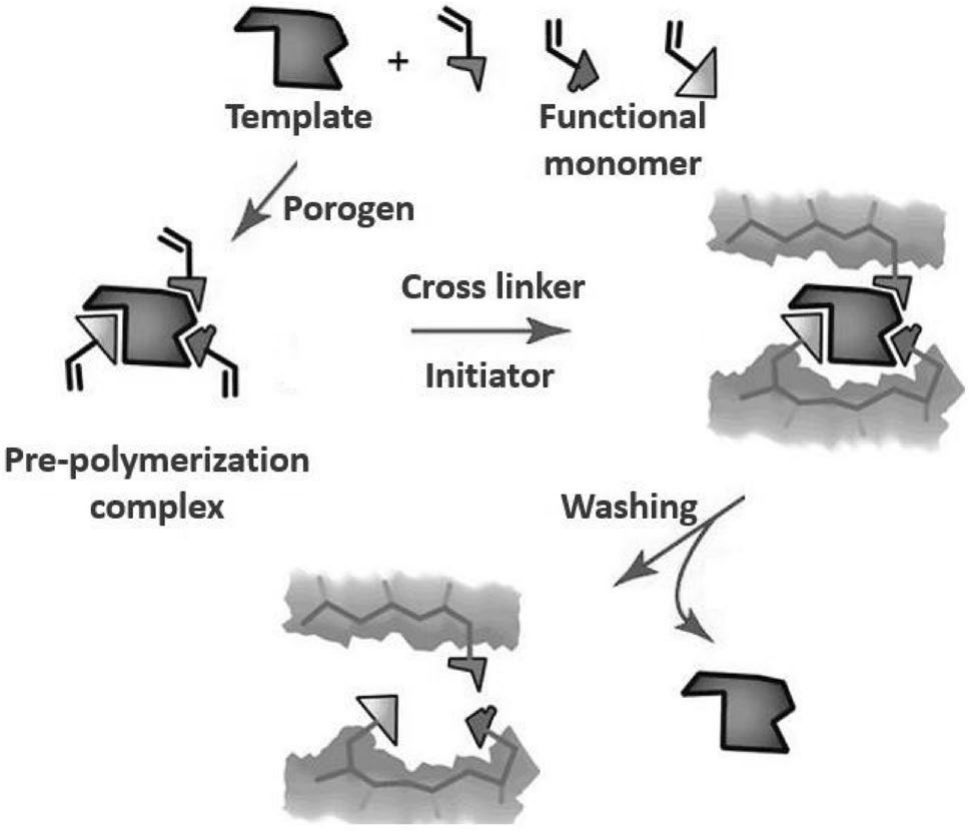
Schematic illustration of MIP preparation using bulk polymerization method

Polymers were prepared following the non-covalent approach via the thermal free radical bulk polymerization method (Electronic Supplementary Material Table S1). In a screw-capped glass tube, 0.2 mmol STZ was dissolved in 4.0 mL of DMF, followed by the addition of the FM (MAA or 4-VP). The mixture was then shaken at room temperature for 15 min for self-assembly of the pre-polymerization complex. Subsequently, the cross-linker EGDMA and 75–90 mg of the AIBN were added. For efficient oxygen gas removal, the solution was then purged with argon for 5 min and left in an oil bath for 24 h at 60 °C (Fig. 2). For each DMIP, a corresponding non-imprinted polymer (NIP) was prepared following the same described procedure but without the addition of STZ [12].

### Removal of the dummy template

For template removal, the resulting polymers were grounded and sieved, and a fraction of 45–100 μm particle size was collected. For template removal, particles were stirred with a 50 mL mixture of DMF/AcOH (9:1, v/v) for an intermittent 8 h (4 h × 2) washing period followed by washing with 100% MeOH for another intermittent 8 h (4 h × 2). Complete removal of the template was monitored using UHPLC-MS/MS.

### Evaluation of binding performance

#### UHPLC-MS/MS method development for STZ quantitation

For UHPLC-MS/MS determination of STZ, a new method was developed where DEXA was used as the internal standard (IS) (Fig. 1). A linear gradient at a flow rate of 0.3 mL min^−1^ was conducted for chromatographic separation using 0.01% FA in water (A) and a 50:50 (v/v) mixture of ACN and MeOH (B). The gradient elution was performed as follows: 40% B (0.0–1.0 min), 40–100% B (1.0–6.0 min), 100% B (6.0–9.0 min), 100–40% B (9.0–10.0 min); then, the concentration of B was kept constant at 40% for 2.0 min for equilibration and column conditioning. Column temperature was set at 45 °C and injection volume was 10 µL performed by partial loop injection using needle overfill as injection technique. As for the ESI-/MS/MS, the mass spectrometer was operated in multiple reaction monitoring (MRM) mode with 4 kV capillary voltage, 300 °C desolvation temperature, and 800 L h^−1^ desolvation flow rate (Table 1).

**Table 1:** MRM transitions, collision energy, and retention time for STZ, DEXA, 3’-OHSTZ, 4β-OHSTZ, and 16β-OHSTZ

Compound	Retention time (min)	MRM transition	Collision energy (V)	Cone voltage (V)
STZ	5.56	329.5 > 81.1	50	125
		329.5 > 121.1	46	
DEXA	4.09	393.1 > 355.2	10	20
		393.1 > 372.19		
3’-OHSTZ	4.67	345.144 > 96.88	44	78
		345.144 > 106.965		
4β-OHSTZ	4.36	345.08 > 309.145	14	34
		345.08 > 327.168		
16β-OHSTZ	4.42	345.144 > 80.936	44	66
		345.144 > 94.991		

#### UHPLC-MS/MS method validation

The method was validated according to the *International Conference on Harmonization* (ICH) guidelines [13]. For linearity, a 5-point calibration curve (5–100 ng mL^−1^) was constructed. Sensitivity was evaluated by finding out the concentration of the STZ where the S/N ratio is 3:1 for the limit of detection (LOD) and 10:1 for the limit of quantitation (LOQ). Quality control (QC) samples of STZ at three different levels, low (7 ng mL^−1^), medium (30 ng mL^−1^), and high (70 ng mL^−1^), were prepared. For intra-day precision, measurement of the QC samples was done on the same day, while for inter-day precision, measurements were done over three consecutive days. Accuracy was evaluated by comparing the practically measured concentration to the theoretical value. The matrix effect was evaluated following the post-extraction spiking method proposed by Matuszewski et al., where the response of the QC samples was compared when spiked post-extraction to their response in a neat solution [14].

#### Batch equilibrium rebinding studies

In Eppendorf**®** Thermomixer compact, 5.0 mg of each polymer was shaken at 750 rpm for 2 h at RT with 2.0 mL of STZ (5 μg mL^−1^) prepared in DMF:MeOH:H_2_O (0.5/0.5/99, v/v/v). This was followed by centrifugation at 14,000 rpm for 10 min and filtration of the resulting supernatants using a 0.22 μm PTFE membrane filter. Supernatants were then fortified with 100 µL of DEXA (5 µg mL^−1^) and the residual amount of STZ was determined using UHPLC-MS/MS. The same experiment was carried out using the same concentration of STZ prepared in DMF:MeOH (0.5/99.5, v/v). All experiments were done in triplicates. Binding capacity (*B*, μmol g^−1^) was calculated using the following Eq. 1 [15];

1 $$B=\frac{({C}_{i}-{C}_{f})\times V\times 1000}{W}$$

*C*_*i*_ and *C*_*f*_ are the initial and final concentrations of the incubated solutions in mM, respectively, *V* is the volume of the solution in mL, and *W* is the weight of the polymer added in mg. The binding capacities of the DMIPs were compared to those of their corresponding NIPs to determine the imprinting factors (*IF*) according to the following Eq. 2 [15];

2 $$IF=\frac{{B}_{MIP}}{{B}_{NIP}}$$

#### Binding isotherm

For measurement of the binding isotherm, a batch of 5.0 mg of the most efficient DMIP and its corresponding NIP was incubated with 2 mL of different initial concentrations (1 µg mL^−1^–20 µg mL^−1^) of the standard STZ prepared in DMF:MeOH (0.5/99.5, v/v), following the same procedures of the batch rebinding studies. All experiments were done in triplicates.

### Characterization of the polymer

FTIR spectra in the range of 4000–500 cm^−1^ were recorded for the powdered STZ, the most efficient DMIP before and after leaching and its corresponding NIP. Surface area, pore volume, and pore size of the polymers were calculated by BET and BJH methods in which the polymers were first degassed at 150 °C for 24 h to remove adsorbed gases and moisture followed by determination of their surface areas by N_2_ adsorption–desorption analysis at 77.3 K. Surface morphology of the best DMIP and its corresponding NIP of particle size (45–100 μm) were examined using SEM.

### DMISPE optimization procedure

DMIP with the best recognition ability for STZ and its corresponding NIP were used to prepare the MISPE. In 3-mL empty frit-containing PTFE SPE cartridges, 10.0 mg of the polymers was separately packed; then, cartridges were conditioned with (3 × 1.0 mL) MeOH and (3 × 1.0 mL) water in turn. This was followed by loading of the tested working solution of STZ, washing of the interferents, and finally elution of the retained analyte. Subsequently, eluates were evaporated for 5 h at 60 °C followed by reconstitution in 1.0 mL MeOH. Analytical measurements of the eluates were done using the developed UHPLC-MS/MS method and % recoveries were calculated. For optimum extraction efficiency of DMISPE, several parameters (loading, washing, and elution conditions and cartridge capacity) were optimized following one factor at a time (OFAT) approach (Electronic Supplementary Material Table S2).

### Application of the optimized DMISP for analysis of STZ metabolites

From a healthy control volunteer, a urine sample was collected and spiked with the appropriate amounts of standard stock solutions of the three STZ metabolites to prepare 60 ng mL^−1^ 3’OHSTZ, 75 ng mL^−1^ 4β-OHSTZ, and 135 ng mL^−1^ 16β-OHSTZ, to acquire concentrations similarly found in the urine after ingesting STZ tablet (20 mg) [3, 16]. Different volumes (1.0 and 2.0 mL) of the spiked urine were loaded. The supernatant was fortified with 100 µL of DEXA (5 µg mL^−1^), followed by evaporation, reconstitution in 550 µL MeOH, filtration, and injection into the UHPLC-MS/MS run under the same previously described gradient elution in “UHPLC-MS/MS method development for STZ quantitation.” For quantitation, the mass spectrometer was operated in MRM mode with 4 kV capillary voltage, 500 °C desolvation temperature, and 1000 L h^−1^ desolvation flow rate (Table 1). The extraction efficiency of STZ metabolites by the optimized DMISPE was evaluated by calculating the extraction *%R* for each metabolite.

## Results and discussion

### Evaluation of binding performance

#### UHPLC-MS/MS method development

**Fig. 3:**
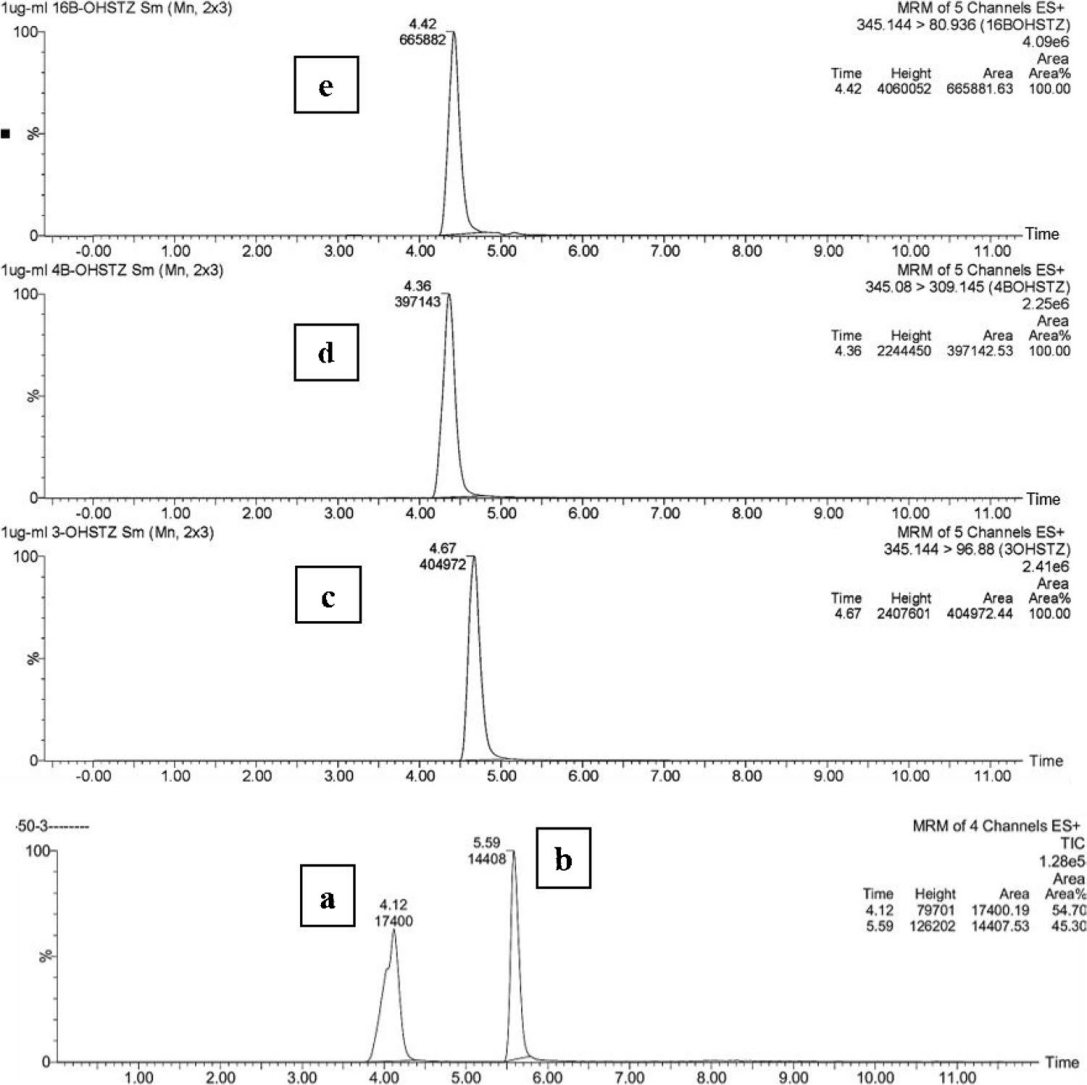
Chromatograms of (**a**) 100 µL of DEXA (5 µg mL^−1^), (**b**) 1 mL of STZ (50 ng mL^−1^) and 1 µg mL^−1^ standard solutions of (**c**) 3’-OHSTZ, (**d**) 4β-OHSTZ, and (**e**) 16β-OHSTZ in MeOH, run under gradient elution at a flow rate of 0.3 mL min^−1^ and a column temperature of 45 °C using 0.01% FA and ACN/MeOH (50:50, v/v) from 40 to 100%B in 5 min

Several methods were reported for the simultaneous determination of STZ and DEXA [17–21], yet none reported the simultaneous determination of STZ together with its 3 main metabolites. Hence, a new UHPLC-MS/MS method was developed to quantify the DMISPE extracted STZ and its main metabolites using DEXA as an internal standard. During the optimization experiments, different percentages (0.001–1%) of FA in the aqueous mobile phase (A) were tested combined with ACN as the organic modifier (B). Best peak symmetry and appropriate retention factor were obtained using a ternary eluent with slight acidic properties namely 0.01% FA in water as the aqueous phase and ACN/MeOH (50:50, v/v) as the organic modifier. Column temperature was set at 45 °C where lower temperature worsened peak resolution and decreased total ion count in the mass detector for both STZ and DEXA. The optimized chromatographic conditions were successfully applied to determine STZ metabolites (Fig. 3).

The ESI interface was operated in the positive mode causing protonation of pyrazole N1 of STZ and its metabolites (Electronic Supplementary Material Fig. S1) [22, 23]. The precursor [M + H]^+^ ion was used in collision-induced dissociation (CID) for MS/MS analysis. The mass spectrometer was operated in MRM mode, where the capillary voltage for all compounds was optimized to 4 kV that resulted in the highest mass response for all the parent ions. Gradual increase of the collision energy optimized the formation of the daughter ions. The MRM channels giving the highest sensitivity and peak area for all compounds were selected for quantitation (Table 1). The fragment ion resulting from the loss of the pyrazole ring from STZ was observed at *m/z* 81.1. As for the metabolites, loss of the pyrazole and 3’-OH from 3’OHSTZ, 2 water molecules from 4β-OHSTZ and the pyrazole ring from 16 β-OHSTZ resulted in the fragment ions observed at *m/z* 96.88, 309.145, and 80.936, respectively [22]. While for DEXA, the loss of HF followed by H_2_O resulted in the fragment ion observed at *m/z* 355.2 [23].

#### UHPLC-MS/MS method validation

**Fig. 4:**
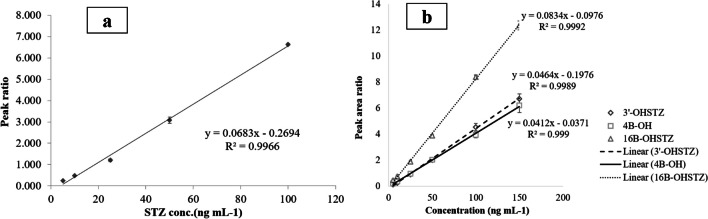
Calibration curve of **a** STZ using DEXA as IS, over the concentration range of 5–100 ng mL^−1^. **b** 3’-OHSTZ, 4β-OHSTZ, and 16β-OHSTZ over the concentration range of 5–150 ng mL^−1^. Each point is the average of 3 measurements and variance is expressed as standard error bars

The developed UHPLC-MS/MS method showed good linearity over the concentration range (5–100 ng mL^−1^) with* R*^*2*^ = 0.9966 (Fig. 4, Electronic Supplementary Material Table S3). Low values of LOD (0.91 ng mL^−1^) and LOQ (1.81 ng mL^−1^) were obtained meeting the MRPL requirements of WADA for STZ (2 ng mL^−1^). The %RSD for precision was below 6%, while the mean *%R* for accuracy was 102.5% ± 1.73 (Table 2). Matrix factors for the 3 QC samples were in the range of 0.93 ± 0.10 and 1.00 ± 0.12 **(**Electronic Supplementary Material Fig S1, S2), which is a tolerable change in the signal response reflecting that there is no matrix effect influencing the analytical measurement of STZ. This implies the highly extractive and clean-up power of the developed DMISPE for STZ [14]**.** Linear calibration graphs were plotted for the three STZ metabolites over the concentration range of (5–150 ng mL^−1^) (*R*^*2*^ > 0.999) (Fig. 4).

**Table 2:** Inter- and intra-day precision and accuracy of the UHPLC-MS/MS for determination of stanozolol (*n* = 3 for each QC sample)

QC sample (ng mL^−1^)	Inter-day precision		Intra-day precision		Accuracy		
	Actual conc. (ng mL^−1^)	%RSD	Actual conc. (ng mL^−1^)	%RSD	Actual conc. (ng mL^−1^)	%Recovery	%RSD
*7*	7.54	7.55	6.92	5.66	7.18	102.68	2.33
*30*	31.51	6.10	32.76	1.97	30.64	102.16	2.67
*70*	72.34	3.64	70.11	0.42	71.86	102.66	0.18

#### Batch equilibrium rebinding study

The popularity and continuous use of the non-covalent bulk polymerization method during MIP synthesis are attributed to the simplicity and cost-effectiveness of this approach. During polymerization in the non-covalent approach, self-assembly of the FM around the T resulted in specific binding sites, while aggregation of the non-associated excess FM resulted in non-specific binding sites. Comparing the binding results of the prepared DMIP with its corresponding NIP highlights the contribution of specific and non-specific binding. A batch rebinding study was done to evaluate the imprinting performance of the prepared polymers, using 2 different rebinding media. When DMF/MeOH/ H_2_O (0.5:0.5:99, v/v/v) was first used as the rebinding medium, the residual supernatant of all polymers consistently resulted in S/N less than 3, indicating that the residual amount of STZ was minimal to be detected. This could be attributed to the low solubility of STZ in water due to its hydrophobic nature which drives it to get drastically adsorbed on all polymers [24]. Accordingly, a rebinding medium free of water (DMF/MeOH (0.5:99.5, v/v)) was used in the batch rebinding studies and B and IF were calculated and summarized in Table 3 and Electronic Supplementary Material Fig S3.

**Table 3:** Binding capacities (*B*) and imprinting factors (IF) of all the prepared polymers in DMF/MeOH (0.5:99.5, v/v)

Polymer	FM	DT:FM:CL	B (µmol g^−1^) ± SD*	IF	*p*-value^a^
DMIP 1	MAA	1:4:20	1.818 ± 0.837	0.749	0.185
NIP 1		0:4:20	2.428 ± 1.012		
DMIP 2		1:4:80	1.644 ± 0.143	0.956	0.345
NIP 2		0:4:80	1.719 ± 0.094		
DMIP 3		1:10:20	1.371 ± 0.782	1.159	0.275
NIP 3		0:10:20	1.183 ± 0.650		
DMIP 4		1:10:80	1.706 ± 0.045	1.317	0.047
NIP 4		0:10:80	1.295 ± 0.185		
DMIP 5	4-VP	1:4:20	2.022 ± 0.745	1.040	0.473
NIP 5		0:4:20	1.944 ± 0.738		
DMIP 6		1:4:80	1.620 ± 0.147	1.117	0.162
NIP 6		0:4:80	1.450 ± 0.006		
DMIP 7		1:10:20	1.172 ± 0.705	0.964	0.483
NIP 7		0:10:20	1.216 ± 0.642		
DMIP 8		1:10:80	1.238 ± 0.162	0.872	0.270
NIP 8		0:10:80	1.419 ± 0.115		

^*^Standard deviation of three measurements

^a^*P*-values were calculated using unpaired, 2-tailed *t*-test

**Fig. 5:**
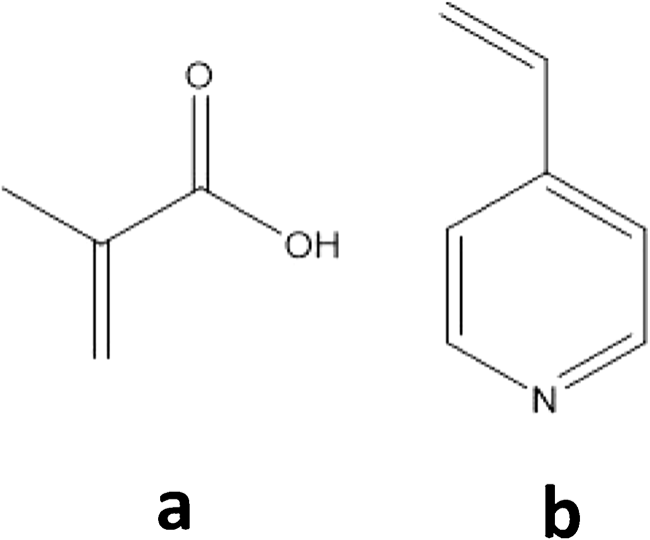
Structures of the functional monomers. **a** Methacrylic acid. **b** 4-Vinylpyridine

Since the interactions between the template and the functional monomer govern the success of the molecular imprinting process, careful choice of functional monomer was one of the most important factors. As per literature, the prevailing type of interaction in the MIPs prepared following the non-covalent approach is hydrogen bonding. Typical functional monomers are carboxylic acids (acrylic acid, methacrylic acid, p-vinylbenzoic acid), sulphonic acids (2 acrylamido-2-methylpropane sulphonic acid), and heteroaromatic bases (4-vinylpyridine, 1-vinylimidazole). MAA has been the most widely used functional monomer for a large variety of template structures due to its capability to act both as a hydrogen bond donor and a hydrogen bond acceptor [25]. The structure of STZ enables it to interact with both, MAA and 4-VP, via π-π interaction and hydrogen bonding (Fig. 5). Higher binding capacities were observed for the MAA polymers in comparison to the 4-VP polymers. This could be explained by the extra ionic interaction taking place between the basic pyrazole ring of STZ and the acidic carboxylic group of MAA which resulted in a more stable pre-polymerization assembly. Generally, in the non-covalent molecular imprinting approach, FM is added in excess relative to the T in order to shift the reaction in the direction of forming T-FM assemblies [26]. For optimizing the amount of MAA functional monomer, both DT:FM ratios of 1:4 and 1:10 were tried and it was observed that polymers with higher molar ratio of MAA showed more specific interaction with the template reflected by the higher values of the IF.

To provide good accessibility for the T to the cavities and stabilize the morphology of the binding sites, optimization of the CL amount was done by testing two different DT:CL ratios of 1:20 and 1:80. In harmony with the literature, findings revealed that DMIPs with higher ratio of EGDMA showed higher IF, confirming that higher degree of stability for the polymers was attained [26]. DMIP# 4 of ratio 1:10:80 (STZ/MAA/EGDMA) showed the highest IF among all the prepared polymers and thus it was the polymer of choice to be used in the subsequent application.

#### Binding isotherm

A binding isotherm was conducted by plotting the bound amount of template (B) against free template concentration (F). The overlaid binding isotherms of DMIP #4 (STZ: MAA: EDGMA, 1:10:80) and its corresponding NIP showed that DMIP #4 had higher binding capacity than its NIP over the initial concentration range of STZ (1 µg mL^−1^–20 µg mL^−1^) (Electronic Supplementary Material Fig S4), verifying that the binding affinity of STZ to DMIP #4 was mainly due to the specific interactions taking place between STZ and the polymer.

### Characterization of DMIP

FTIR is widely used in the assessment and characterization of imprinted products. The similarity in the FTIR spectra of the leached DMIP# 4 and its corresponding NIP indicates that both polymers have a similar backbone (Electronic Supplementary Material Fig S5 a, b). The FTIR results showed a broad (–OH) stretching peak at ~ 3446 cm^−1^ for DMIP and ~ 3447 cm^−1^ for NIP corresponding to the (–COOH) in MAA. The (–CH_2_) stretching peak due to the methyl and methylene groups existing in the polymer network was also observed at ~ 2957 cm^−1^ in DMIP and ~ 2958 cm^−1^ in NIP. The strong spike at ~ 1732 cm^−1^ in DMIP and ~ 1733 cm^−1^ in NIP corresponds to the (–C = O) stretching in EGDMA and MAA. Finally, the observed bands at ~ 1150 cm^−1^ in DMIP and ~ 1151 cm^−1^ in NIP correspond to the (–C–O) bending bands in EGDMA and MAA. The spectra of STZ and unleached DMIP# 4 highlighted the successful incorporation of STZ into the polymer (Electronic Supplementary Material Fig S5 c, d). The band observed at 1656 cm^−1^ in STZ and 1667 cm^−1^ in the unleached DMIP spectra corresponds to the (–C = N) stretching of the pyrazole ring in STZ. Additionally, the (–N–H) stretching band of the pyrazole ring of STZ was observed at ~ 3357 cm^−1^ in the STZ spectrum, yet it was partially masked by the broad (–OH) stretching band in the unleached DMIP# 4 spectrum. Successful template removal during the washing step was confirmed by the disappearance of the (–C = N) stretching band of STZ in both the NIP and the leached DMIP# 4 spectra.

**Fig. 6:**
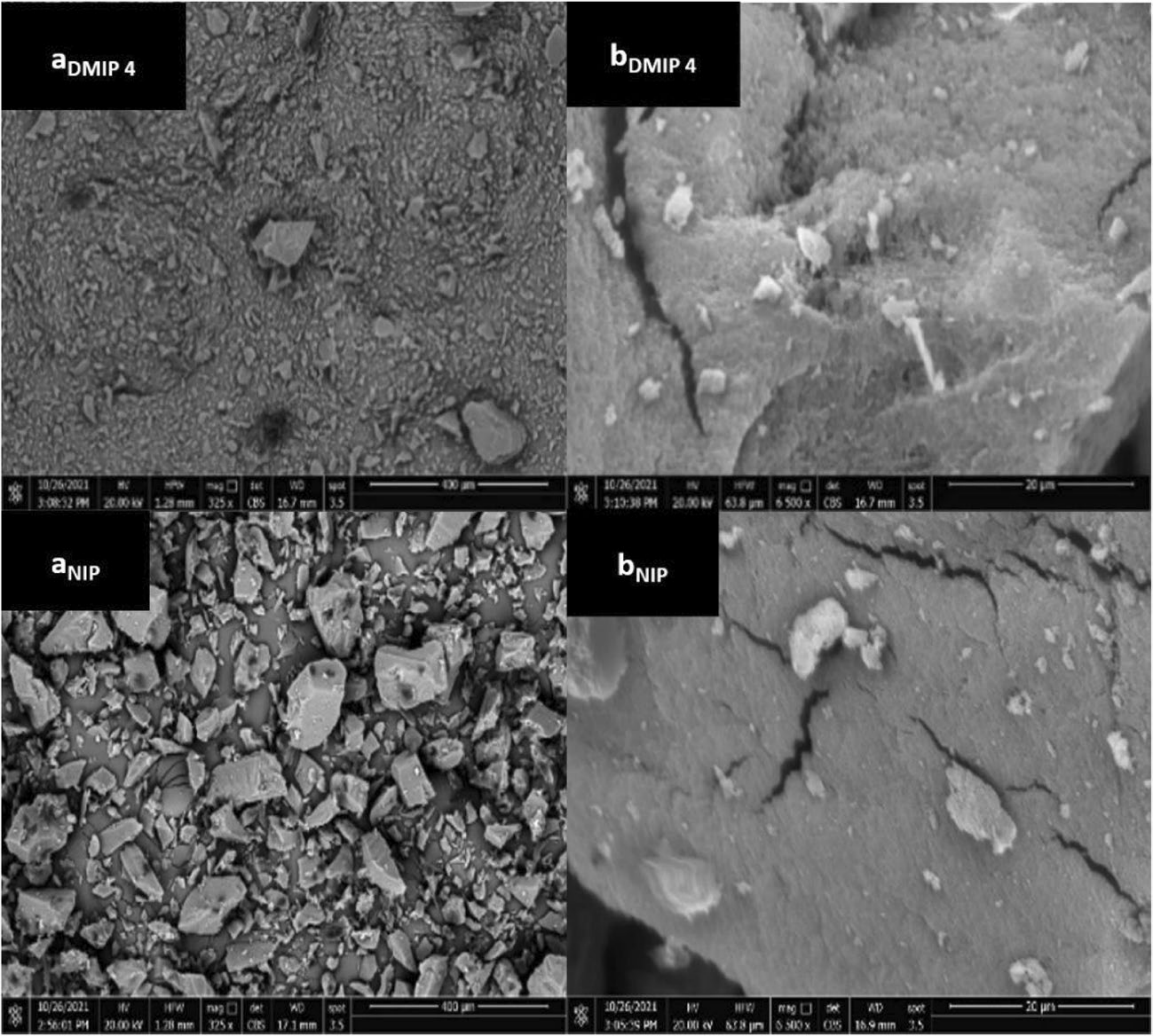
SEM images of DMIP#4 (STZ:MAA:EGDMA, 1:10:80) and NIP#4, at (**a**) × 325 and (**b**) × 6500 magnification powers

The derived BET and BJH data displayed that DMIP# 4 exhibited lower surface area and pore volume (23.38 m^2^ g^−1^, 0.07 cc g^−1^) than its corresponding NIP (58.36 m^2^ g^−1^, 0.16 cc g^−1^) (Electronic Supplementary Material Table S4). This could be explained by the impact of conducting the free radical polymerization process at low temperatures, which as per *Sellergren*, would bring about unfavorable changes in the morphology of the polymer resulting in low pore volume and surface area [27]. Moreover, BJH results revealed that DMIP# 4 has a mesoporous structure with pore radii ranging from 1.86 to1.94 nm, which makes DMIP# 4 a potential sorbent to be used in SPE, since the mesoporous structures are more permeable for solvents compared to micropores and do not require the application of high pressure. From the view of the aforementioned findings, it can be concluded that the binding performance of DMIP# 4 can be mainly attributed to the specifically tailored cavities during the imprinting process rather than the morphology of the polymer [28]. The SEM images of DMIP# 4 and its NIP show irregularity of the particle surfaces caused by the nature of the bulk polymerization method used in their preparation, which is in agreement with the previous reports for bulk polymers (Fig. 6) [29].

### DMISPE optimization procedure

#### Optimization of the loading solvent

Sample loading is an important step in DMISPE to allow selective retention of the target analytes with high amounts. Loading step optimization started using similar conditions of the previously described rebinding study, namely the concentration of STZ (5 μg mL^−1^), sorbent amount (10.0 mg), and rebinding medium composition (DMF/MeOH (0.5:99.5, v/v)). Results showed that most of the drug was recovered in the loading fraction (Electronic Supplementary Material Table S2), resulting in low % recoveries in the elution fraction (elution *%R*). This outcome was initially hypothesized to result from oversaturating the sorbent sites with the drug [24]. So as a trial to increase the number of the available binding sites for STZ rebinding, 2 sets of trials were done, where lower concentrations of STZ (2.5 μg mL^−1^) and larger amounts of the polymer (15.0, 20.0 mg) were tested. However, no considerable improvement in the elution *%R* was observed (~ 12.6% ± 2.13) (Electronic Supplementary Material Table S2)*.*

**Fig. 7:**
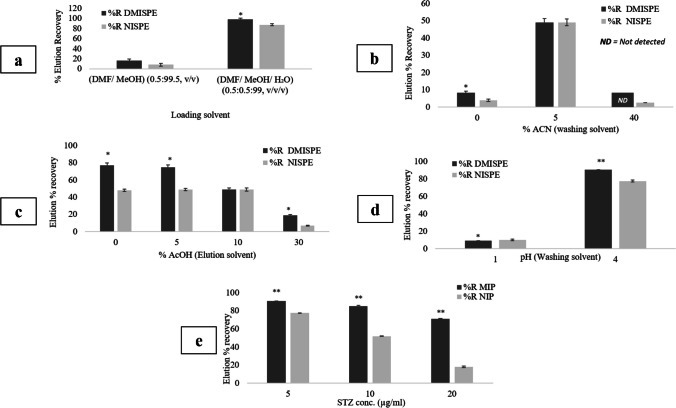
Percentage recoveries obtained from DMISPE and NISPE during optimizing different SPE parameters: **a** loading solvent, **b** % ACN in washing solvent, **c** % AcOH in elution solvent, **d** pH of washing solvent, and **e** cartridge capacity

The loading medium is essential, since it directly affects the recognition ability of MIP, taking into account the type of T-FM interactions. Hence, the loading solvent composition was modified, where a significant proportion of water was added (DMF/MeOH/H_2_O (0.5:0.5:99, v/v/v). It was observed that substantial improvement in the elution *%R* occurred (98% ± 4.06) and this was further confirmed by percolating through the cartridge different STZ concentrations** (**Fig. 7, Electronic Supplementary Material Table S2). This upswing in the elution *%R* could be interpreted by the poor solubility of STZ in water which maximizes the adsorption of STZ on the polymer in the loading step [30].

#### Optimization of the washing solvent

Ideal washing solvent is the selective solvent which is capable of reducing the non-specific interaction at the binding sites, eluting the contaminants that may interfere with the targeted analyte, while providing high specific retention for the analyte [31]. Optimization of the washing step started using pure water as the washing solvent and results revealed that a significant loss of STZ (~ 50%) in the washing fraction occurred due to the highly polar protic nature of water which is capable of disrupting the hydrogen bond interaction taking place between STZ and the polymer [32] (Fig. 7, Electronic Supplementary Material Table S2).

Literature reports mixture of ACN/water as a commonly used washing solvent is SPE [33]. Accordingly, two sets of trials were carried out by adding intermediate (40%) and low levels (5%) of ACN to the aqueous washing solvent. Results showed a remarkable improvement in the elution *%R* (~ 50% ± 2.20) when an aqueous solution of 5% ACN was used, indicating that adding the less polar aprotic ACN to the aqueous washing solvent decreases the disruption of the interaction between STZ and DMIP, thus stabilizing and maintaining the specific interaction of STZ with the binding sites [34]. It was also observed that the detection of STZ was almost not feasible in the SPE fractions of both DMISPE and NISPE when a higher level of ACN was added. This may imply that STZ retention was non-specifically maximized on the polymers to the extent that it became almost non-recoverable in any of the SPE fractions (Fig. 7, Electronic Supplementary Material Table S2). Based on these findings, H_2_O/ACN (95:5, v/v) was selected to be the washing solvent of choice, yet up to this point, it is worth mentioning that no imprinting effect was observed.

#### Optimization of the elution solvent

Efficient elution of the target compounds is achieved by disrupting the interaction between the retained analytes and the sorbent surface [32]. MeOH is a polar protic solvent capable of interfering with the hydrogen bond interactions taking place between STZ and the polymer resulting in its elution. According to the dissociation constants of STZ (pKa_1_ = 3.36 and pKa_2_ = 16.23) [35], it was hypothesized that adding AcOH would result in protonation of the pyrazole nitrogen, diminishing its ability to hydrogen bond with MeOH and thus decreasing the elution strength of MeOH. To test for this, different %AcOH in MeOH (0, 5, 10, 20, 30%) were tested. Findings revealed that using methanol without any added acid resulted in a significant improvement of STZ recovery in the elution fraction which was pronounced in the imprinting effect (DMISPE = 77% ± 3.99, NISPE = 48 ± 2.01) (Fig. 7, Electronic Supplementary Material Table S2). This reflects that pure MeOH as an elution solvent was capable of suppressing the specific binding and to a lesser extent the non-specific binding resulting in higher specific recovery of STZ from the DMISPE [31], making it the solvent of choice for further experiments.

#### Optimization of the pH of washing solvent and evaluation of the cartridge capacity

According to the dissociation constants of STZ, it can be present in the ionized and unionized form; accordingly, its retention in the SPE cartridges could be pH-dependent. To study this effect, cartridges were percolated with 5% ACN solutions of different pH (1.0 and 4.0) using acetate buffer (25 mM). In a strongly acidic medium (pH = 1.0), ionization of STZ took place increasing its solubility in the polar ACN/water mixture which subsequently resulted in its loss in the washing fraction (elution *%R* = 9.00% ± 0.21) (Fig. 7, Electronic Supplementary Material Table S2). On the contrary, using a washing solvent of pH 4.0 resulted in the preservation of the drug in its unionized limited solubility form thus stabilizing STZ interaction with the polymer during the washing step. This resulted in minimal loss of the drug during washing while significantly improving the elution *%R* (DMISPE 90.94% ± 0.04, NISPE 77.69% ± 0.02, *p*-value = 0.00032), Accordingly, 5% ACN in acetate buffer (25 mM, pH 4.0) was selected to be the washing solvent.

It is worth noting that the IFs and the binding isotherm reported in this study resulted from a static rebinding study, which just gives a preliminary selection for the best polymer as a start off point for the rest of the optimization experiments. Nevertheless, a more obvious significant difference in the performance between the optimized DMIP and its corresponding NIP has been highlighted in the SPE experiments where the optimized DMISPE resulted in a higher elution %R for the STZ compared to its corresponding DNISPE, as mentioned above.

Using the optimized DMISPE method, cartridge capacity was evaluated by percolating 1 mL of different STZ concentrations (5, 10, and 20 μg mL^−1^) prepared in DMF:MeOH:H_2_O (0.5:0.5:99, v/v/v%) through 10.0 mg polymer. It was found that lower recovery of the drug in the elution fraction was observed when higher STZ concentration was loaded (Fig. 7, Electronic Supplementary Material Table S2), which could be explained by oversaturation of the polymer binding sites resulting in poor recoveries of the drug in the elution fractions [24, 36].

### Application of DMISPE for analysis of STZ metabolites

**Fig. 8:**
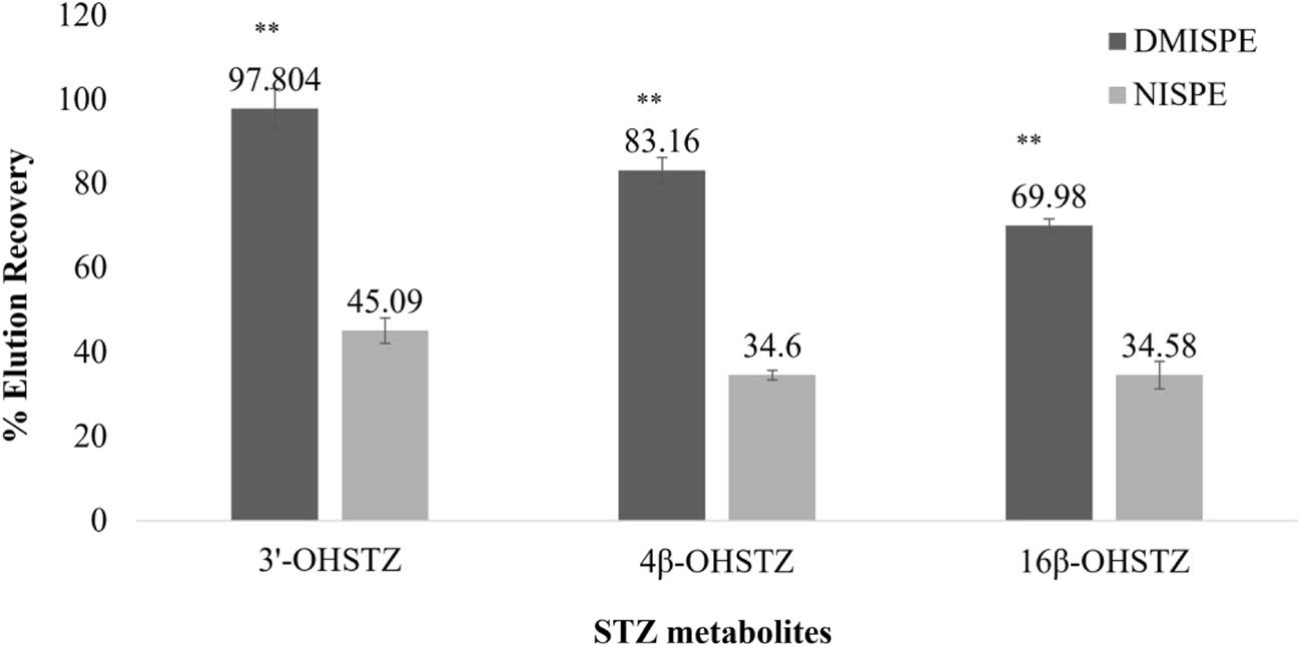
Recoveries of 3’-OHSTZ (60 ng mL^−1^), 4β-OHSTZ (75 ng mL^−1^), and 16β-OHSTZ (135 ng mL^−1^) from spiked human urine samples after application of the optimized DMISPE and NISPE

**Fig. 9:**
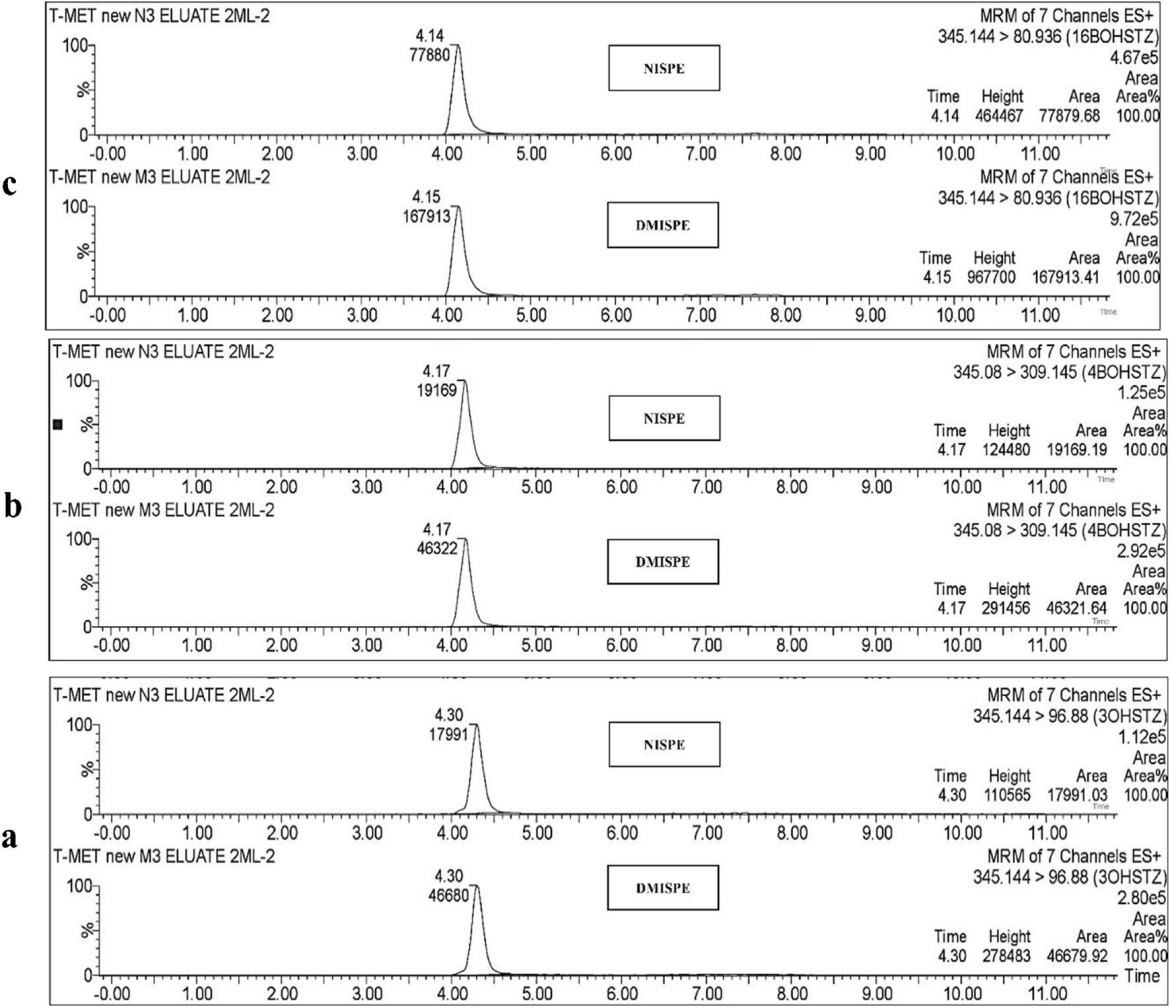
Chromatograms of extracted metabolites from urine sample **a** 3’-OHSTZ spiked at 135 ng mL^−1^, **b** 4β-OHSTZ spiked at 75 ng mL^−1^, and **c** 16β-OHSTZ spiked at 60 ng mL.^−1^

As a proof of concept, for evaluating the efficiency of the optimized DMISPE in extracting the three main metabolites from a human urine sample, 1 mL urine was spiked with 3’-OHSTZ, 4β-OHSTZ, and 16β-OHSTZ at concentrations of 60, 75, and 135 ng mL^−1^, respectively. Results showed that for all metabolites, poor extraction recovery was observed (55.60%, 35.80%, and 34.13% for 3’-OHSTZ, 4β-OHSTZ, and16β-OHSTZ respectively). As per the literature, extraction efficiency might be improved by increasing the loading volume, as this would allow for better interaction between the polymer and the analyte resulting in increasing the amount of the target analyte adsorbed on the surface of the sorbent [37]. Accordingly, a higher loading volume (2 mL) was tested and as expected significant improvement in the extraction recovery for all metabolites was observed (99.80%, 83.16%, and 69.98% for 3’-OHSTZ, 4β-OHSTZ, and 16β-OHSTZ respectively, *P*-values ≤ 0.005) (Figs. 8 and 9, Electronic Supplementary Material Table S5). The variable extraction recovery between the three metabolites could be attributed to the variable positions of the phase 1 metabolism hydroxyl group. This positioning leads to variable interaction strengths with the optimized DMIP.

## Conclusion

To the best of our knowledge, this is the first study reporting the use of STZ for the preparation of DMIP which was applied as an SPE sorbent for simultaneous extraction of STZ metabolites from human urine samples. Results revealed that higher extraction recovery of the three metabolites was obtained from the optimized DMISPE (69.98–97.80%) compared to its NISPE (34.58–45.09%). Owing to the highly extractive and clean-up power of the newly developed DMISPE method, better sensitivity (LOD = 0.91 ng mL^−1^) was observed compared to other reported UPLC-MS/MS method (LOD = 10.00 ng mL^−1^) which used LLE as its sample pre-treatment method [38]. This makes the developed DMISPE a promising clean-up method to be used by the regulatory anti-doping control labs.

### Supplementary Information

Below is the link to the electronic supplementary material.

Supplementary file1 (DOCX 680 KB)

Supplementary file2 (XLSX 26 KB)

## Data Availability

The data supporting the findings of this research are available within the article and its supplementary materials.

## Declarations

### Source of biological material

One urine sample was collected from one healthy volunteer from laboratory staff.
