# Supplementary material for: Novel dummy molecularly imprinted polymer for simultaneous solid-phase extraction of stanozolol metabolites from urine
Source: Anal Bioanal Chem. 2024 Apr 25;416(14):3335–47. doi: 10.1007/s00216-024-05285-x (PMC11106188; doi:10.1007/s00216-024-05285-x)
Supplement: Supplementary file 1 — Supplementary file1 (DOCX 680 KB) [file 216_2024_5285_MOESM1_ESM.docx]

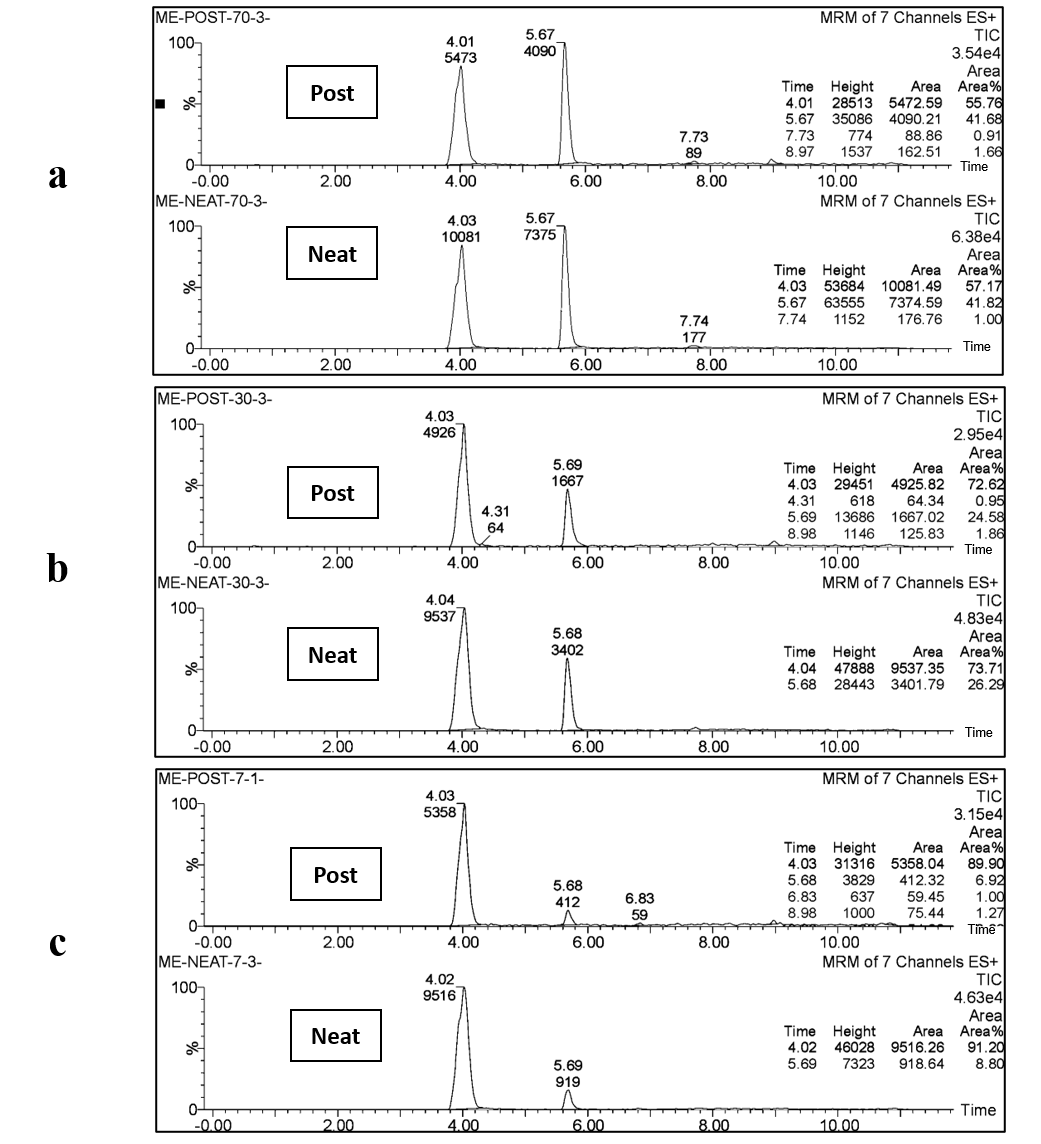


Fig. S1 Chromatograms of QC samples of STZ (t_R_~5.68) with 100 µL of (5 µg ml^-1^) DEXA (t_R_~4.03) in neat solution (neat) and post extraction spiked urine (post), where a, b and c are the 3 concentration levels of 7,30,70 ng ml^-1^ of STZ respectively.


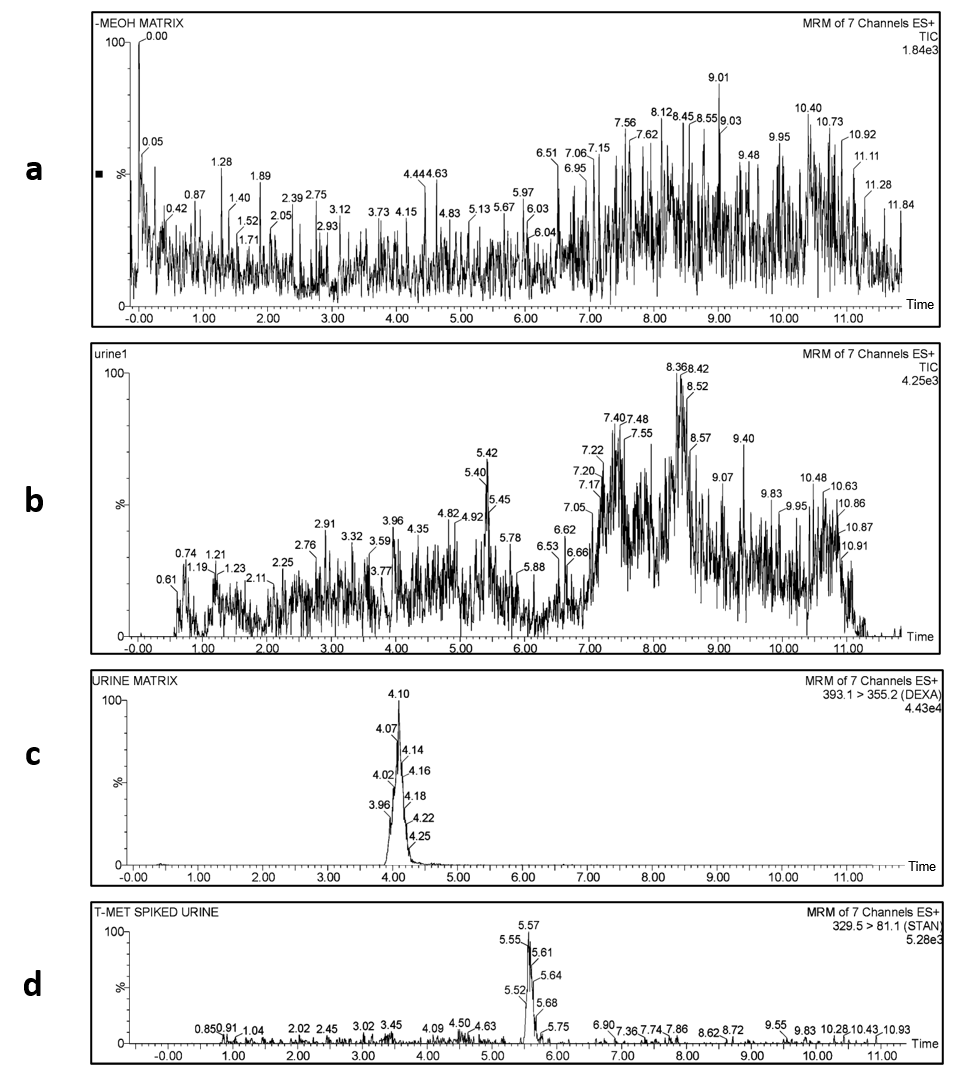


**Fig. S2** Chromatograms of **(a):** Blank MeOH, **(b):** Blank urine, **(c):** DEXA (IS) added to a blank urine and **(d):** 50 ng mL^-1^ STZ added to a blank urine.


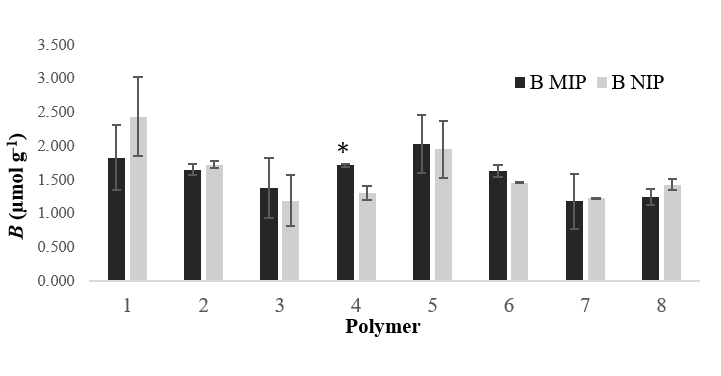


Fig. S3 Binding capacities of all DMIPs and their corresponding NIPs (readings are mean of 3 measurements ± RSD).

**Fig. S4** Binding isotherm of DMIP# 4 (STZ: MAA: EGDMA, 1:10:80) and its corresponding NIP (readings are average of three measurements ± SD).


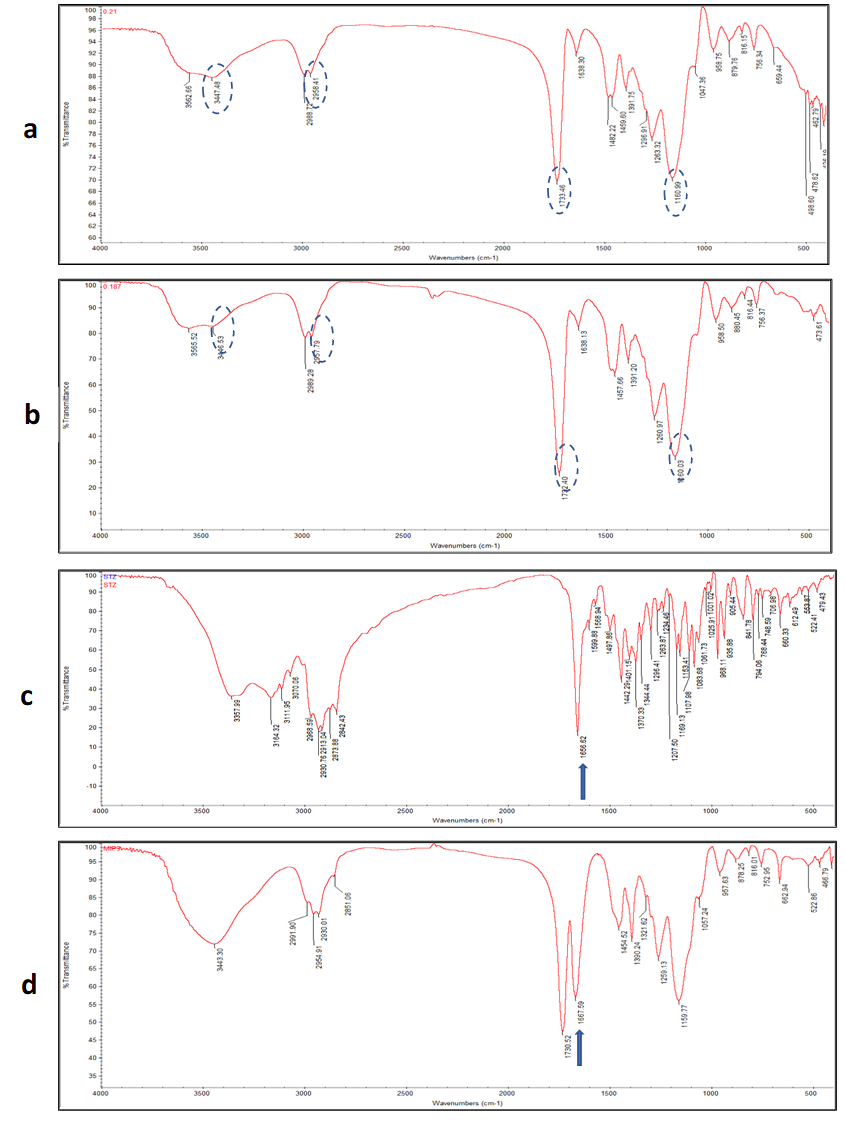


Fig. S5 FT-IR spectra for (a) NIP 4, (b) leached DMIP# 4, (c) STZ and (d) unleached DMIP# 4 (STZ: MAA: EGDMA, 1:10:80)
